# Collateral benefits of studying the vagus nerve in bioelectronic medicine

**DOI:** 10.1186/s42234-019-0021-3

**Published:** 2019-05-16

**Authors:** Valentin A. Pavlov

**Affiliations:** 1 Center for Biomedical Science and Bioelectronic Medicine, The Feinstein Institute for Medical Research, Northwell Health System, Manhasset, NY 11030 USA; 2 Donald and Barbara Zucker School of Medicine at Hofstra/Northwell, Hempstead, NY 11550 USA

**Keywords:** Vagus nerve, Bioelectronic medicine, Cholinergic, Inflammation, Cytokines, Inflammatory diseases

## Abstract

Studies on the role of the vagus nerve in the regulation of immunity and inflammation have contributed to current preclinical and clinical efforts in bioelectronic medicine. In parallel, this research has generated new insights into the cellular and molecular mechanisms underlying the immunoregulatory functions of the vagus nerve within *the inflammatory reflex*. The vagus nerve and other cellular components of the inflammatory reflex are implicated in the regulation of bleeding, cancer, obesity, blood pressure, viral infections and other conditions. This *collateral benefit* broadens scientific horizons and provides new rationale for technological advances and therapeutic implications.

## Background

The vagus nerve has an essential role as a conduit of information between the brain and the periphery and in maintaining physiological homeostasis and defense mechanisms. During the last 20 years we have witnessed an explosion of new information substantially extending the knowledge about the regulatory functions of the vagus nerve. The recent discovery of the role of the vagus nerve in the regulation of immunity and inflammation within *the inflammatory reflex* is a major contribution to this new information (Borovikova et al., 2000; Tracey, 2002). Inflammation is a vital immune defense mechanism against invading pathogens, tissue damage and other immunological threats. Inflammation is tightly regulated to restore homeostasis (Nathan, 2002). However, inflammation does not always follow this normal scenario. Several forms of excessive, unresolved and chronic inflammation manifest and mediate pathogenesis in sepsis, rheumatoid arthritis, inflammatory bowel disease (IBD) and other inflammatory and autoimmune conditions (Nathan, 2002; Pavlov et al., 2018). Even obesity and cancer pathogenesis importantly involves immune dysregulation and aberrant inflammation (Pavlov & Tracey, 2017; Balkwill & Mantovani, 2001). Therefore, controlling inflammation is critically important in preventing and treating many conditions and diseases.

**Fig. 1:**
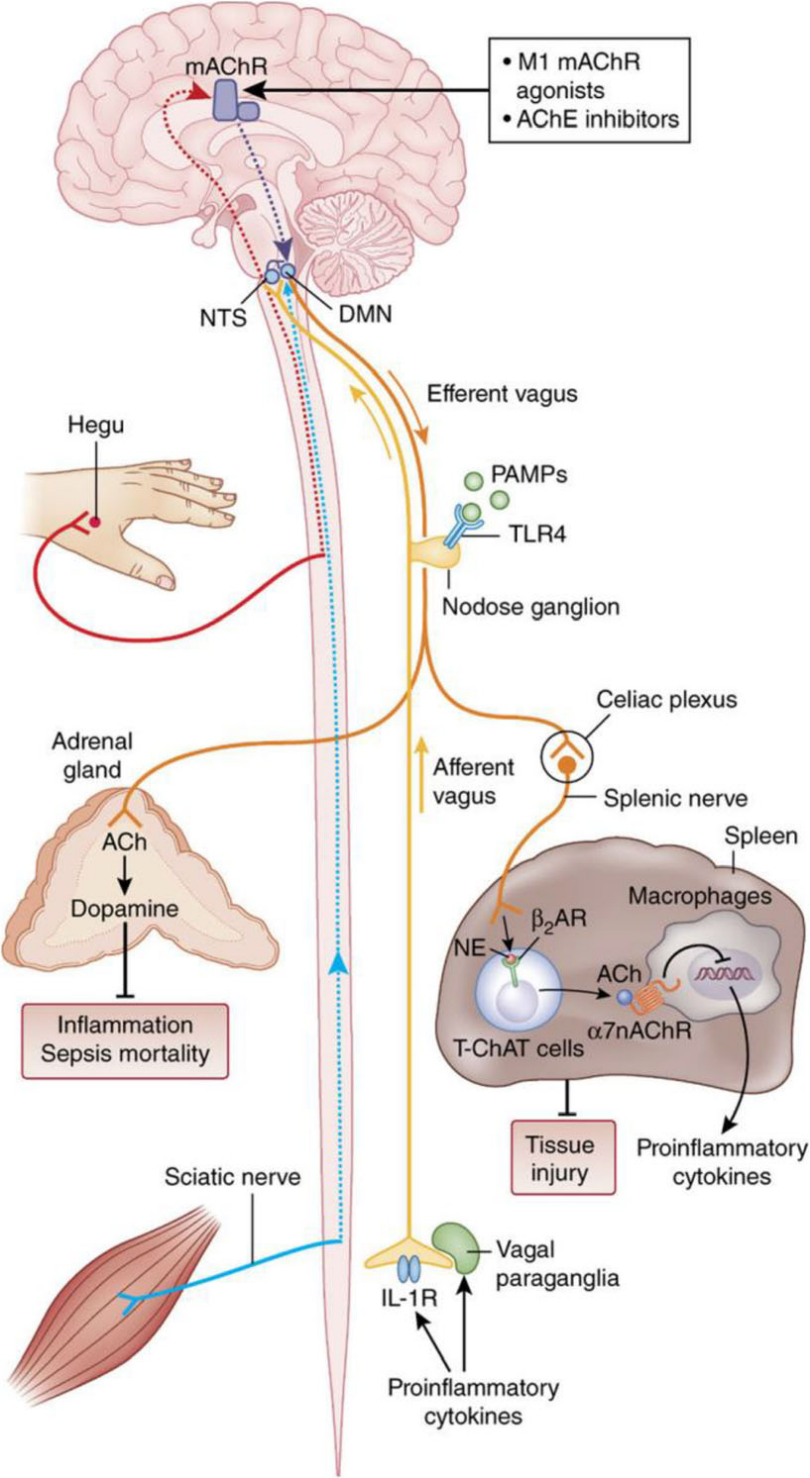
The inflammatory reflex. In the inflammatory reflex, the activity of afferent vagus nerve fibers residing in the nodose ganglion is stimulated by cytokines and pathogen-associated molecular patterns (PAMPs). The signal is transmitted to the NTS. Reciprocal connections between the NTS and DMN mediate communication with and activation of efferent vagus nerve fibers from the DMN. The signal is propagated to the celiac ganglia and the superior mesenteric ganglion in the celiac plexus, where the splenic nerve originates. Norepinephrine (NE) released from the splenic nerve interacts with β_2_-adrenergic receptors (β_2_-ARs) and causes the release of acetylcholine (ACh) from T cells containing functional choline acetyltransferase (T-ChAT cells). ACh interacts with α7nAChRs on macrophages and suppresses proinflammatory cytokine release and inflammation. The inflammatory reflex can be activated through brain mAChR-mediated mechanisms by centrally-acting M1 mAChR agonists and acetylcholinesterase (AChE) inhibitors. Somatosensory activation by electroacupuncture at the Hegu point also causes activation of brain mAChR signaling, which then results in activation of efferent vagus and splenic anti-inflammatory signaling. Electroacupuncture at a different acupuncture point activates sciatic nerve signals, which by unknown mechanisms convert to efferent vagus nerve signaling to the adrenal medulla, resulting in dopamine release. Dopamine suppresses inflammation and improves survival in a model of sepsis. Vagus nerve and splenic nerve signaling mediated through α7nAChR on splenocytes controls inflammation in acute kidney injury and alleviates the condition. *(Figure created by Debbie Maizels, Springer Nature, for Pavlov and Tracey 2017; reprinted, with permission, from the authors in conjunction with Springer Nature)*

**Fig. 2:**
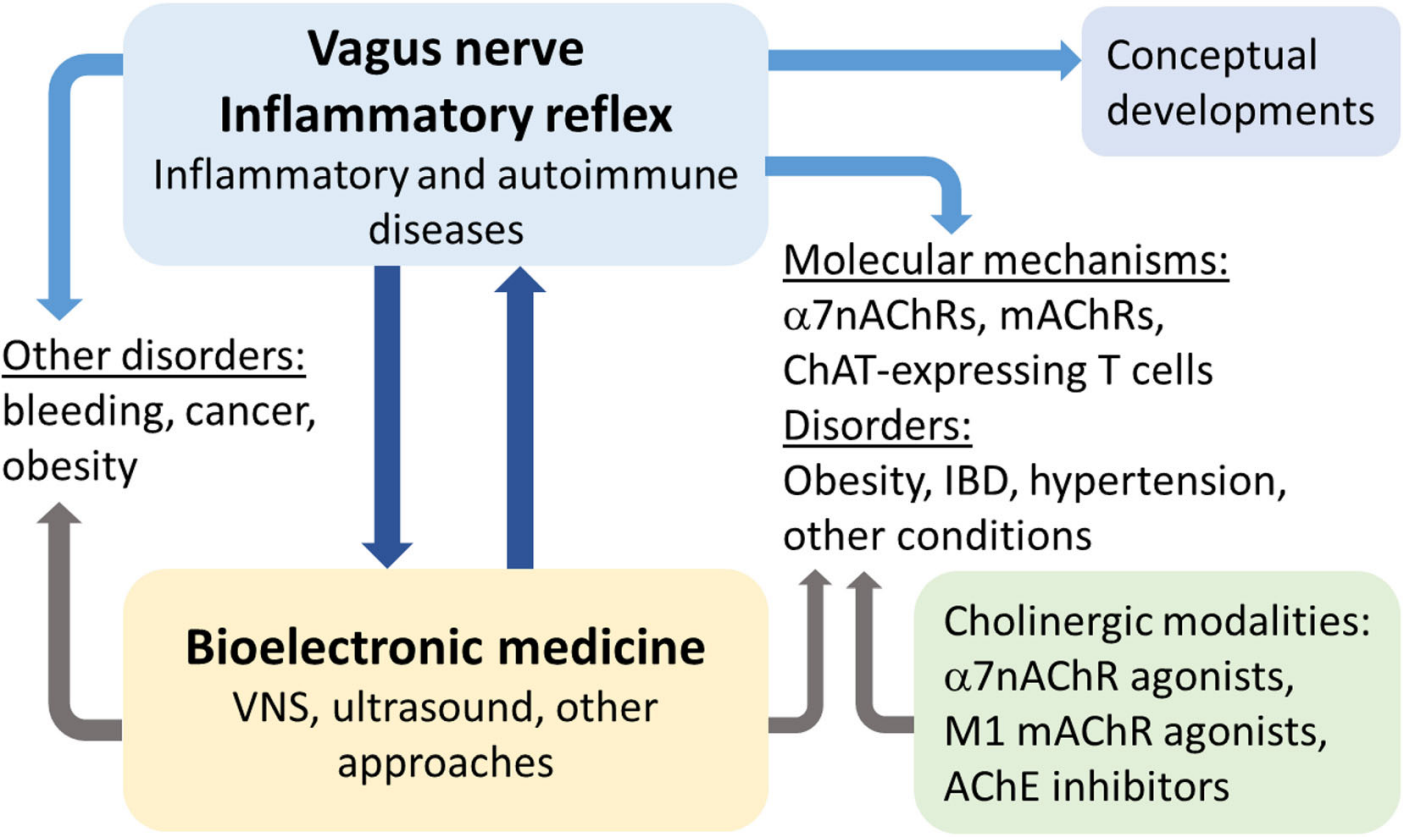
The immunoregulatory functions of the vagus nerve in bioelectronic medicine and associated insights and advances. Preclinical studies on the immunoregulatory role of the vagus nerve and the inflammatory reflex in the context of inflammatory and autoimmune diseases have resulted in successful clinical trials exploring implanted device-generated VNS in Crohn’s disease and rheumatoid arthritis under the umbrella of bioelectronic medicine. There is a symbiotic relationship between these and other ongoing clinical studies and active preclinical research. Studies stemming from this research have identified vagus nerve regulatory functions in bleeding, cancer, obesity and other disorders, which can also be targeted for therapeutic benefit by bioelectronic approaches. Molecular and cellular mechanisms underlying the inflammatory reflex involve α7nAChRs, brain mAChR and ChAT-expressing T lymphocytes. Their role as molecular therapeutic targets has been indicated in obesity-associated disorders, IBD (colitis), hypertensive and hypotensive conditions and other disorders, which can be targeted for therapeutic benefit by α7nAChR agonists, centrally-acting M1 mAChR agonists and AChE inhibitors and bioelectronics. All of this research, technological advances and therapeutic approaches have been accompanied by charting new relevant concepts and their ongoing validation (See text for details)

Recent studies demonstrated the important role of the vagus nerve in controlling pro-inflammatory cytokine release and inflammation within the inflammatory reflex (Tracey, 2002; Pavlov & Tracey, 2017) **(**Fig. 1**)**. The anti-inflammatory and disease-alleviating efficacy of electrical vagus nerve stimulation (VNS) in numerous animal models of inflammatory disease have been described. This abundant knowledge provided a rationale for studying the therapeutic utility of bioelectronic VNS in human inflammatory and autoimmune diseases **(**Fig. 2**)**. Recent successful clinical trials with implanted device-generated VNS in patients with rheumatoid arthritis, IBD and other conditions have validated the efficacy of this approach (Bonaz et al., 2016; Koopman et al., 2016). Both preclinical and clinical research on the anti-inflammatory function of the vagus nerve have contributed to current development in bioelectronic medicine **(**Fig. 2**)**. This growing field utilizes new research insights into the regulatory functions of the nervous system and technological advances in the development of novel diagnostic and treatment approaches for a broad spectrum of diseases and conditions (Pavlov et al., 2018; Pavlov & Tracey, 2019). In parallel with streamlining the studies on the anti-inflammatory functions of the vagus nerve in the context of bioelectronic medicine, considerable insights into the mechanisms underlying these functions have been generated. Moreover, the scope of disorders in which VNS or cholinergic modalities can be applied for therapeutic benefit has been extended. New discoveries related to the broader physiological role of cellular constituents of the vagus nerve-based inflammatory reflex have also been made. This research improves understanding of neural regulation, presents new therapeutic avenues both for bioelectronic medicine and other fields, leads to conceptual developments, and advances science as a whole. Here, I briefly summarize the role of the vagus nerve in the neuro-immune dialogue with relevance to bioelectronic medicine, and focus on the broader scope of new insights generated, designating them as *collateral benefits*.

### The vagus nerve: neurophysiological considerations

The vagus nerve is a mixed nerve comprised of afferent (sensory, about 80%) and efferent (motor, about 20%) neurons. Vagus nerve afferent neurons are pseudounipolar cells residing in the nodose ganglia (Berthoud & Neuhuber, 2000). The peripheral axonal terminals of these neurons in many visceral organs sense fluctuations in metabolic and cardiovascular homeostasis and this information is communicated to the brainstem nucleus tractus solitarius (NTS) where central axon endings terminate (Berthoud & Neuhuber, 2000; Chavan et al., 2017; Pavlov & Tracey, 2012). Neural communication between NTS and the dorsal motor nucleus of the vagus (DMN) mediates brainstem integration of afferent and efferent vagus nerve signaling, because DMN is a major source of efferent (motor) vagus neurons. These long preganglionic cholinergic neurons communicate with short postganglionic neurons in close range to or within the lungs, heart, pancreas and other innervated organs. Acetylcholine released from these neurons interacts with metabotropic muscarinic acetylcholine receptors (mAChRs) on smooth muscle cells, cardiac myocytes and glandular cells and regulates a broad spectrum of physiological functions including bronchoconstriction, heart rate, gastrointestinal motility and secretion and pancreatic endocrine and exocrine secretion (Pavlov & Tracey, 2012).

### The vagus nerve in the neuro-immune dialogue: from preclinical studies to bioelectronic medicine

Studies initiated about 20 years ago substantially broadened knowledge about the vagus nerve by revealing the role of this nerve in the regulation of immunity and inflammation (Borovikova et al., 2000; Tracey, 2002). This discovery complemented earlier studies demonstrating the role of afferent vagus nerve fibers in the gastrointestinal tract, hepatic portal system and other visceral organs in monitoring peripheral immune milieu (Chavan et al., 2017). These studies have demonstrated that afferent vagus neurons communicate signals about peripheral levels of IL-1β, TNF and other inflammatory molecules to the brain and specifically to NTS (Fig. 1). Innovative research on the role of afferent and efferent vagus nerve signaling in the context of inflammation led to a major conceptual development – integrated afferent and efferent vagus nerve signaling regulates immune responses and inflammation within *the inflammatory reflex (Tracey,* 2002*)* (Fig. 1). Electrical vagus nerve stimulation (VNS) was used to discover the role of the efferent vagus nerve in controlling the levels of TNF and other pro-inflammatory cytokines (Pavlov & Tracey, 2015). In addition, acetylcholine, a major mediator of efferent vagus nerve signaling, suppresses endotoxin-activated macrophage release of TNF, IL-1β, and other pro-inflammatory cytokines (Borovikova et al., 2000). Numerous studies in rodent endotoxemia (Borovikova et al., 2000), sepsis (Huston et al., 2006), post-operative ileus (de Jonge et al., 2005), collagen-induced arthritis (Levine et al., 2014), colitis (Meregnani et al., 2011), and other conditions have indicated that VNS can be used as a therapeutic approach to alleviate aberrant inflammation (Pavlov & Tracey, 2015).

Insight from these ongoing pre-clinical studies recently led to the first clinical trials in patients with inflammatory disorders, including IBD (Crohn’s disease) (Bonaz et al., 2016) and rheumatoid arthritis (Koopman et al., 2016). These preclinical and clinical studies accelerated the growing field of bioelectronic medicine (Pavlov et al., 2018; Pavlov & Tracey, 2019) **(**Fig. 2**)**. The first clinical trials utilized implanted devices for VNS that had already been clinically-approved for the treatment of epilepsy and depression (Bonaz, 2018). Encouraging results from the clinical trials generated parallel efforts focused on technological development, aimed at miniaturizing, improving the control and optimizing the therapeutic regimens of electrodes and devices (Levine et al., 2019). In parallel, development and testing of devices and approaches for non-invasive VNS in pre- and clinical settings and generating relevant mechanistic insight is also underway. For instance, non-invasive transcutaneous auricular VNS has been recently shown to activate NTS to DMN neural interactions and suppress inflammation in preclinical endotoxemia and postoperative ileus (Hong et al., 2018). In humans, the same type of stimulation has been shown to cause NTS and other brain neuronal activation patterns consistent with the “classical” afferent vagus nerve projections (Frangos et al., 2015). The therapeutic efficacy of transcutaneous auricular vagus nerve stimulation in depression (Kong et al., 2018) and other forms of non-invasive and invasive VNS in treating chronic pain has been actively studied (Chakravarthy et al., 2015). In addition to VNS, the anti-inflammatory and therapeutic benefit of other approaches, including electrical acupuncture and ultrasound, activating neural circuitry within the inflammatory reflex was demonstrated in preclinical models of sepsis, acute kidney injury, and endotoxemia (Pavlov & Tracey, 2017; Inoue et al., 2016; Torres-Rosas et al., 2014; Cotero et al., 2019). (Figs. 1, 2). In parallel, substantial insight into the cellular and molecular constituents of the inflammatory reflex considerably broadened the horizons for therapeutic developments.

### Revealing the mechanisms

A somewhat surprising receptor mechanism underlies the anti-inflammatory role of the vagus nerve. It turned out that in contrast to many “classical” physiological functions, mediated through metabotropic mAChRs, the vagus nerve anti-inflammatory effects are mediated through ionotropic nicotinic acetylcholine receptors (nAChRs) (Wang et al., 2003). A specific role for a subset of these receptors, containing the α7 subunit and expressed on immune cells in this regulation (Figs. 1, 2) was revealed through several observations. VNS fails to significantly alter serum TNF levels in mice genetically deficient for the α7nAChR during endotoxemia (Wang et al., 2003). In addition, while cholinergic treatment with nicotine significantly suppresses pro-inflammatory cytokine release by macrophages it fails to generate this effect in macrophages from α7nAChR KO mice or macrophages with α7nAChR anti-sense downregulation (Wang et al., 2003). Furthermore, α7nAChR expression in bone marrow-derived immune (non-T cells) was shown to play an essential role for the anti-inflammatory effect of VNS (Olofsson et al., 2012). A detailed understanding of intracellular mechanisms mediating α7nAChR-dependent cholinergic suppression of pro-inflammatory cytokine release has emerged. These mechanisms involve inhibition of the nuclear factor kappa-light-chain-enhancer of activated B cells (NF-κB) nuclear translocation (Guarini et al., 2003; Parrish et al., 2008), activation of the Janus kinase 2 - signal transducer and activator of transcription 3 (JAK2-STAT3) pathway (de Jonge et al., 2005), inhibition of the inflammasome (Lu et al., 2014), and inhibition of adenylyl cyclase 6 (Tarnawski et al., 2018).

The inflammatory reflex is also regulated centrally (in the brain) and several studies have revealed a role for mAChRs and specifically the M1 mAChR in this regulation in endotoxemia, inflammatory bowel disease (colitis), hemorrhagic shock and other conditions (Pavlov & Tracey, 2015; Pavlov et al., 2006; Munyaka et al., 2014; Lee et al., 2010) (Fig. 1). Enhancing brain cholinergic signaling through administering centrally-acting acetylcholinesterase inhibitors such as galantamine also results in suppression of aberrant inflammatory responses through vagus nerve-mediated signaling in mouse models of endotoxemia, colitis and lupus (Pavlov et al., 2009; Ji et al., 2014; Pham et al., 2018). A very recent study utilizing selective optogenetic stimulation and advanced pharmacological approaches indicated a specific role for forebrain cholinergic signaling and M1 mAChR in the inhibition of peripheral inflammatory responses via vagus nerve signaling in murine endotoxemia (Lehner et al., 2019).

VNS results in suppression of splenic TNF, which has been identified as a substantial contributor to systemic TNF. How the vagus nerve controls cytokines in the spleen is noteworthy. The vagus nerve innervates the celiac ganglia and the superior mesenteric ganglion – a documented source of neurons within the splenic nerve (Pavlov & Tracey, 2015). The endings of these catecholaminergic neurons release predominantly norepinephrine, which modulates T lymphocytes, which contain choline acetyltransferase (ChAT), the enzyme responsible for the synthesis of acetylcholine (Rosas-Ballina et al., 2011). These ChAT-expressing CD4^+^ T lymphocytes play an important role in mediating the TNF-suppressing effect of the vagus nerve (Rosas-Ballina et al., 2011) (Figs. 1, 2). VNS increases splenic acetylcholine levels via splenic nerve catecholaminergic signaling acting on lymphocytes, and VNS fails to significantly suppress TNF levels in mice, lacking T cells (nude mice), during endotoxemia. In addition, passive adoptive transfer of ChAT-expressing T cells into nude mice restores the anti-TNF effect of VNS (Rosas-Ballina et al., 2011). Essential involvement of the vagus nerve-splenic nerve link has been indicated in neural regulation of immunity in endotoxemia (Rosas-Ballina et al., 2011), murine colitis (Munyaka et al., 2014; Ji et al., 2014), T cell activation and egress from the spleen, and the regulation of hypertension (Carnevale et al., 2016), renal ischemia–reperfusion injury (Inoue et al., 2016), and other conditions (Pavlov & Tracey, 2015).

### The collateral scientific and therapeutic benefit

The intriguing role of ChAT-expressing T cells as a source of acetylcholine in the neural circuitry of the inflammatory reflex importantly implicated these cells in the regulation of innate immunity and inflammation. This discovery generated new research efforts that provided insights extending the scope of ChAT-expressing T cell functional repertoire in physiological and disease contexts. The physiological regulation of blood pressure is complex and involves several brain-orchestrated and peripheral neuro-endocrine and metabolic components. Acetylcholine is importantly involved in blood pressure regulation and the vasorelaxant properties of this molecule related to the release of nitric oxide are well documented. However, the source of acetylcholine in this context has remained somewhat enigmatic. Further characterization of the distinct T-cell population defined by ChAT identified them as CD4^+^CD44^hi^CD62L^lo^ T helper cells by gene expression (Olofsson et al., 2016). Mice with selective deficiency of ChAT expression in CD4^+^ cells have higher arterial blood pressure as compared to littermate control mice, and infusion of Jurkat T cells overexpressing ChAT significantly lowers blood pressure (Olofsson et al., 2016). In addition, co-incubation of these Jurkat T cells and endothelial cells increases phosphorylation of endothelial nitric oxide synthase, and nitrate and nitrite levels in conditioned media, suggesting activated release of nitric oxide – a molecule with a key role in vasolelaxation (Olofsson et al., 2016). These findings demonstrate the important role of CD4^+^ T_ChAT_ cells in blood pressure regulation. They provide a rationale for further studies on these cells in hypotension and hypertension, and designing new therapeutic approaches focused on cell-mediated vasorelaxation (Olofsson et al., 2016) (Fig. 2). Another very recent study revealed the important role of lymphocyte-released acetylcholine in the regulation of chronic viral infections. Lymphocytic choriomeningitis virus (LCMV) infection is associated with significantly increased ChAT levels in both CD4^+^ and CD8^+^ T cells in an IL-21-dependent manner (Cox et al., 2019). Mice with selective *Chat* deficiency within the T cell compartment have impaired vasodilation in response to infection, decreased migration of antiviral T cells into infected tissues, and substantially compromised control of chronic LCMV clone 13 infection. These findings represent a genetic proof of function for ChAT in T cells during viral infection and implicate ChAT-expressing T cells in antiviral immunity (Cox et al., 2019).

The growing insight into the inflammatory reflex and the receptor mechanisms mediating efferent vagus nerve anti-inflammatory output prompted new clinically-oriented research. The anti-inflammatory and disease-alleviating effects of many α7nAChR agonists, including nicotine, GTS-21, choline, and PNU-282987 have been demonstrated in preclinical settings of sepsis, postoperative neuroinflammation, ischemia-reperfusion injury, obesity and type 2 diabetes, and other disorders (Parrish et al., 2008; Pavlov et al., 2007; Chatterjee et al., 2017; Mavropoulos et al., 2017; Terrando et al., 2011; Wang et al., 2011; Li et al., 2013; Yeboah et al., 2008; Hoover, 2017; Terrando & Pavlov, 2018) **(**Fig. 2**)**. Centrally-acting mAChR agonists and acetylcholinesterase inhibitors have shown promising efficacy in murine models of sepsis, inflammatory bowel disease, obesity, lupus, and other conditions (Munyaka et al., 2014; Ji et al., 2014; Pham et al., 2018; Rosas-Ballina et al., 2015; Satapathy et al., 2011; Yang & Yang, 2018; Hanes et al., 2015) **(**Fig. 2**)**. One of these centrally-acting acetylcholinesterase inhibitors – galantamine is a clinically-approved drug for the treatment of Alzheimer’s disease. This fact, the abundant information about the safety profile of this drug and the rationale from preclinical studies (Satapathy et al., 2011) facilitated performing a recent clinical trial with this drug in patients with the metabolic syndrome (Consolim-Colombo et al., 2017). In this randomized placebo-controlled trial, treatment with clinically-approved doses of galantamine significantly alleviated the inflammatory state and insulin resistance and modulated the autonomic neural function towards vagus nerve predominance (Consolim-Colombo et al., 2017).

Another line of research emerging from studying the role of the vagus nerve in immune regulation identified a new role of this nerve in the regulation of bleeding (hemorrhage) (Czura et al., 2010) (Fig. 2). VNS in pigs subjected to ear resection significantly shortens the bleeding time and increases the thrombin/antithrombin III complex formations (Czura et al., 2010). These findings suggest that VNS can be further explored for therapeutic benefit in hemorrhage-related conditions.

An accumulating body of data also reveals an intriguing role for the vagus nerve in preclinical scenarios of cancer, including breast, gastric and pancreatic cancer (Chavan et al., 2017; Pavlov & Tracey, 2015) **(**Fig. 2**)**. Two recent studies provide important insights into the role of the vagus nerve and cholinergic signaling in pancreatic exocrine cancer. In mice with pancreatic ductal adenocarcinoma, subdiaphragmatic vagotomy results in increased tumor growth and worsened survival (Partecke et al., 2017). This is associated with increased TNF levels and increased number of tumor associated macrophages. In addition, the survival of TNF knockout mice implanted with the tumor is significantly longer (Partecke et al., 2017). In another study subdiaphragmatic vagotomy in mice with LSL-*Kras* ^+/G12D^; *Pdx1*-Cre model of this cancer significantly accelerates tumorigenesis, whereas activation of cholinergic signaling via administration of a mAChR agonist has suppressive effects (Renz et al., 2018). Stimulated cholinergic signaling specifically results in suppression of the cancer stem cell compartment, CD11b^+^ myeloid cells, TNF levels, and metastasis in the liver (Renz et al., 2018). These findings suggest the potential of exploring VNS and cholinergic modalities in the treatment of such an aggressive disease with notoriously vague symptoms as pancreatic exocrine cancer (Fig. 2).

Chronic low-grade inflammation has a documented role in promoting insulin resistance and other metabolic derangements in obesity and obesity-related conditions, including the metabolic syndrome, type 2 diabetes, cardiovascular disease and non-alcoholic steatohepatitis (Pavlov & Tracey, 2012). There is a growing interest in exploring vagus nerve circuitry by using bioelectronics in the treatment of the modern pandemics of obesity and obesity-related disorders by exploring vagus nerve efferent anti-inflammatory signaling and vagus nerve afferents with a role in satiety (Pavlov & Tracey, 2012; Yao et al., 2018) (Fig. 2).

Research on the immunomodulatory function of the vagus nerve and its brain regulation also contributed significantly to formulating the concepts of *the immunological homunculus *(Pavlov et al., 2018; Tracey, 2007), the *set point function of the immune responses *(Tracey, 2009), *the neuroimmune communicatome *(Olofsson et al., 2017; Metz & Pavlov, 2018), and *innate immune exhaustion* in sepsis survivors (Zaghloul et al., 2017) (Fig. 2). Ongoing and future studies utilizing advanced technics for molecular mapping and selective modulation of neural circuitry will undoubtedly provide new insight within the framework of these conceptual models and indicate new therapeutic avenues (Pavlov & Tracey, 2017; Chang et al., 2015; Chang, 2019).

## Conclusions

Improved understanding of the role of the vagus nerve in controlling inflammation and innovative applications of this knowledge led to current clinical developments in treating inflammatory and autoimmune diseases with implantable bioelectronics. These advances benefited parallel technological developments related to improving these devices and testing new devices for non-invasive bioelectronic VNS (Levine et al., 2019; Mertens et al., 2018). In parallel, other approaches within the scope of bioelectronic medicine, including electrical acupuncture and ultrasound for activating circuitries in the inflammatory reflex have showed promising therapeutic efficacy **(**Fig. 2**)**. Demonstrating a role for the vagus nerve in the control of bleeding, cancer, and obesity, and the important involvement of a subset of ChAT-expressing T cells in the regulation of blood pressure and very recently – in viral infection are all important examples of discoveries stemming from studying the immunoregulatory role of the vagus nerve. Revealing the molecular mechanisms of efferent vagus nerve anti-inflammatory control have indicated the therapeutic applicability of α7nAChR agonists and centrally-acting acetylcholinesterase inhibitors, with first examples of clinical validation in clinical settings **(**Fig. 2**)**. These developments represent a broad spectrum of collateral benefit of innovative research on the vagus nerve with current clinical implications in bioelectronic medicine **(**Fig. 2**)**. Multidisciplinary collaborative research efforts will continue to advance bioelectronic medicine and benefit other disciplines with the goal of creating therapeutic approaches that will make a difference for our patients.

## Abbreviations

ACh: Acetylcholine
AChE: Acetylcholinesterase
ChAT: Choline acetyltransferase
DMN: Dorsal motor nucleus of the vagus
IBD: Inflammatory bowel disease
IL-1: 1βinterleukin-1β
IL-21: Interleukin-21
JAK2-STAT3: Janus kinase 2- signal transducer and activator of transcription 3
LCMV: Lymphocytic choriomeningitis virus
mAChRs: Muscarinic acetylcholine receptors
nAChRs: Nicotinic acetylcholine receptors
NE: Norepinephrine
NF-κB: Nuclear factor kappa-light-chain-enhancer of activated B cells
NTS: Nucleus tractus solitarius
PAMPs: Pathogen-associated molecular patterns
TNF: Tumor necrosis factor
VNS: Vagus nerve stimulation
β_2_-ARs: β_2_-adrenergic receptors
